# A combined radio-immunotherapy regimen eradicates late-stage tumors in mice

**DOI:** 10.3389/fimmu.2024.1419773

**Published:** 2024-07-15

**Authors:** Alexander L. Rakhmilevich, Noah W. Tsarovsky, Mildred Felder, Jen Zaborek, Sritha Moram, Amy K. Erbe, Alexander A. Pieper, Dan V. Spiegelman, Emily M. Cheng, Cole M. Witt, Willem W. Overwijk, Zachary S. Morris, Paul M. Sondel

**Affiliations:** ^1^ Department of Human Oncology, University of Wisconsin-Madison, Madison, WI, United States; ^2^ Department of Biostatistics and Medical Informatics, University of Wisconsin-Madison, Madison, WI, United States; ^3^ Nektar Therapeutics, San Francisco, CA, United States; ^4^ Department of Pediatrics, University of Wisconsin-Madison, Madison, WI, United States

**Keywords:** cancer immunotherapy, radio-immunotherapy, late-stage tumors, combination (combined) therapy, mouse tumor models

## Abstract

**Background:**

The majority of experimental approaches for cancer immunotherapy are tested against relatively small tumors in tumor-bearing mice, because in most cases advanced cancers are resistant to the treatments. In this study, we asked if even late-stage mouse tumors can be eradicated by a rationally designed combined radio-immunotherapy (CRI) regimen.

**Methods:**

CRI consisted of local radiotherapy, intratumoral IL-12, slow-release systemic IL-2 and anti- CTLA-4 antibody. Therapeutic effects of CRI against several weakly immunogenic and immunogenic mouse tumors including B78 melanoma, MC38 and CT26 colon carcinomas and 9464D neuroblastoma were evaluated. Immune cell depletion and flow cytometric analysis were performed to determine the mechanisms of the antitumor effects.

**Results:**

Tumors with volumes of 2,000 mm^3^ or larger were eradicated by CRI. Flow analyses of the tumors revealed reduction of T regulatory (Treg) cells and increase of CD8/Treg ratios following CRI. Rapid shrinkage of the treated tumors did not require T cells, whereas T cells were involved in the systemic effect against the distant tumors. Cured mice developed immunological memory.

**Conclusions:**

These findings underscore that rationally designed combination immunotherapy regimens can be effective even against large, late-stage tumors.

## Introduction

During the last decade, cancer immunotherapy has become a major pillar of cancer treatment and has shown remarkable efficacy in certain cancers (1–3). To evaluate new immunological treatments, mouse models with relatively small transplantable or spontaneous tumors are commonly used. Large tumors are typically resistant to immunotherapy and therefore first need to be debulked either by surgery or chemotherapy to see efficacy (4–6). A systematic analysis by Wen et al. (7) of experimental immunotherapies on tumors differing in size showed that a vast majority of animal studies use small tumors, and regression of tumors larger than 200 mm^3^ was rare and observed mainly after certain combinatorial approaches or adoptive T cell therapy. Based on these and similar observations, it is generally believed that immunotherapy (other than adoptive T cell therapy) is effective only against relatively small tumors and tumor metastases. Often, when antitumor effects are reported against large subcutaneous tumors, they are generally defined as large or late-stage when their volumes are less than 200 mm^3^. For example, some authors define large tumors as equal or more than 5 mm in diameter or ~60 mm^3^ in tumor volume (8), and others define large, late-stage tumors having diameters of ~7x8 mm or ~200 mm^3^ (9). In our previous study, we observed that a combination of local radiotherapy (RT), slow-release pegylated interleukin 2 (srIL-2) and antibody against cytotoxic T-lymphocyte antigen 4 (CTLA-4) induced regression of 1,000 mm^3^ B78 melanomas in some mice (10). We asked if mice with even larger tumors can be cured with this or a similar combinatory approach. We hypothesized that adding interleukin 12 (IL-12), a cytokine which has been shown to synergize with IL-2 (11–13), would improve the efficacy of our treatment combination. In this study, using several tumor models, we show that an approach combining local RT, srIL-2, IL-12 and anti-CTLA-4, which we call a combined radio- immunotherapy (CRI) regimen, can eradicate tumors with volumes of 2,000 mm^3^ or larger. Future optimization of this regimen, e.g., minimizing potential adverse effects, is warranted for clinical use.

## Material and methods

### Study design

The primary objective of this study was to test the hypothesis that rationally designed CRI will induce regression of late-stage solid tumors in several mouse tumor models. All tumor efficacy experiments were performed with at least five mice per experimental group. Before the first treatment, mice were randomized to achieve similar mean tumor volumes in each group. The treatments and tumor measurements were performed in blind fashion. The experiments were reproduced one or more times to substantiate the results. The differences in survival between treatment groups were estimated between days 75 and100 after the beginning of treatment.

### Mice

Female 7–12 week-old C57BL/6 and BALB/c mice were obtained from Taconic Farms (Germantown, NY). Mice were housed in the University of Wisconsin-Madison animal facilities at the Wisconsin Institutes for Medical Research. Mice were used in accordance with the *Guide for Care and Use of Laboratory Animals* (NIH publication 86-23, National Institutes of Health, Bethesda, MD, 1985). Experiments were performed under an institutional animal care and use committee approved animal protocol. Mice were euthanized when the tumor diameter reached 20 mm or when mice became moribund. Mice were euthanized by carbon dioxide inhalation.

### Tumor cell lines

Mouse B78-D14 (B78) melanoma is syngeneic to C57BL/6 mice; it is a slow growing derivative of B16 melanoma that has been transfected to express GD2, and it was obtained from Dr. Ralph Reisfeld (14). MC38 and CT26 (ATCC) are mouse adenocarcinomas syngeneic to C57BL/6 and BALB/c mice, respectively. Mouse 9464D neuroblastoma syngeneic to C57BL/6 mice was obtained from Jon Wigginton, MD, while at the National Cancer Institute (NCI), Bethesda, MD. GD2-expressing clone, 9464D-GD2 Cl.7.2, was made in our lab as described previously (15). The tumor cell lines were grown in RPMI-1640 (B78 and MC38) or DMEM (CT26 and 9464D-GD2) cell culture medium supplemented with 10% FBS (Sigma-Aldrich, St. Louis, MO), 2 mM L-glutamine, and 100 U/ml penicillin/streptomycin at 37°C in a humidified 5% CO2 atmosphere. Mycoplasma testing via PCR was routinely performed.

### *In vivo* tumor models

Intradermal (i.d.) tumors were established by injecting 2x10^6^ (B78 and 9464D-GD2) or 1x10^6^ (MC38 and CT26) cells in 0.1 ml of PBS into the shaved flank (16).

Tumor diameters were measured, and tumor volume (mm^3^) was calculated as [½ x tumor length x tumor width^2^]. One day before RT, mice were randomized according to tumor volumes and assigned to experimental groups. For depletion experiments, anti-CD4, clone GK1.5 (200 *μ*g), anti-CD8, clone 2.43 (100 *μ*g), anti-NK1.1., clone PK136 (100 *μ*g) or rat IgG (300 *μ*g) was given via intraperitoneal (i.p.) injection on d. -1, 6, 13, 20, and 27 of the experiment. The depleting antibodies were purchased from Bio X Cell, and rat IgG was purchased from Sigma Aldrich. For rechallenge experiments, mice that rejected B78, MC38 or CT26 tumors following CRI, 2-4 months later were rechallenged i.d. with 2x10^6^ B78 cells, 1x10^6^ MC38 cells or 1x10^6^ CT26 cells, respectively, at the shaved flank opposite from the initial tumor. Naïve mice were injected with the same tumor cells as control.

### Reagents

Anti-CTLA-4 clone 9D9, IgG2c isotype, was obtained from Bristol-Myers Squibb, Redwood City, CA (17). SrIL-2, Bempegaldesleukin, is a CD122-preferential IL-2 receptor agonist that consists of recombinant human IL-2 conjugated with an average of six releasable molecules of polyethylene glycol and thus achieves slow release of IL-2 *in vivo* circumventing the limitations of traditional high dose systemic IL-2 immunotherapy such as dose-limiting toxicity and a short *in vivo* half-life (18). SrIL-2 was provided by Nektar Therapeutics. Mouse recombinant IL-12 was purchased from BioLegend.

### RT

RT of flank tumors using an X-Rad 320 irradiator (Precision X-Ray, Inc.) was performed as previously described (19). Mice were immobilized using custom-designed lead jigs which exposed right flank tumor to irradiation and shielded normal tissues and untreated distant tumors. RT (300kVp voltage and 10mA current) was administered in one fraction delivering 12 Gy. The day of RT was defined as day 0 of treatment.

### Immunotherapy

Tumor-bearing mice were treated with intravenous (i.v.) injections of 16 *μ*g srIL-2 in 0.1 ml of buffer on days 5, 14, and 23 (18). IL-12 (0.5 *μ*g in 0.1 ml PBS) was injected intratumorally (i.t.) on days 5-7. Anti-CTLA-4 (0.2 mg in 0.2 ml PBS) was injected i.p. on days 2, 5 and 8. Administering cytokines starting on day 5 days after RT was based on our previous publication (19). CRI included srIL-2, IL-12, anti-CTLA-4, and RT.

### Flow cytometry of tumor-infiltrating cells

C57BL/6 mice bearing advanced B78 melanoma or MC38 adenocarcinoma were treated with CRI or RT (control) as described above. On day 8 or 12, tumors were harvested, cut into small pieces, and incubated for 30 minutes at 37°C in dissociation solution containing RPMI supplemented with 5% FBS, 1mg/ml Collagenase type D and 100µg/ml DNase I (Sigma-Aldrich, St. Louis, MO), using a Miltenyi gentleMACS Octo Dissociator. Samples were then incubated with Tonbo Ghost Dye Violet 510 (Cytek 13-0870-T500, 1µL/test) for 30 min at 4°C, followed by washing in flow buffer (PBS-1% FBS) and a 10-minute incubation with anti-CD16/32 Tonbo FC Shield (Cytek 70-0161-U500, Clone 2.4G2, 1µL/test) at 4°C. Cells were surface stained in Brilliant Stain Buffer (BD Biosciences) for 30 minutes at 4°C with CD45 FITC (BioLegend 103108, clone 30-F11, 0.5µl/test), CD25 BB700 (BD Biosciences 566498, clone PC61, 2µL/test), CD8a APC-Fire 750 (BioLegend 100766, clone 53-6.7, 1µL/test), and CD4 BV785 (BioLegend 100453, clone GK1.5, 3µL/test). Samples were washed, then fixed and permeabilized for 30 minutes at 4°C with the FoxP3/Transcription Factor Staining Buffer Set from eBioscience (Invitrogen 00-5523-00) following kit instructions. Samples were then stained with FoxP3 PE-Cy7 (Invitrogen 25-5773-82, clone FJK-16s, 2µL/test) from eBioscience for 30 minutes at room temperature. All samples were then washed in permeabilization buffer and resuspended in flow buffer for acquisition on an Attune flow cytometer (ThermoFisher). UltraComp eBeads (ThermoFisher 01-2222-42) were used for fluorophore compensation and data were analyzed with FlowJo v10 software (BD). Debris was excluded on FSC vs SSC plots, dead cells were excluded via Ghost Dye positivity, and doublets were excluded via FSC-H vs FSC-W plots. Remaining events were considered to be live, single cells and were gated for CD45+ before proceeding with further gating of immune populations; FMO samples were used to establish negative gates for each immune cell marker. Data were analyzed with FlowJo v10 software (BD). Tregs were defined as live, single, CD45+ CD4+ CD25+ FoxP3+ cells. Statistical analysis was performed using GraphPad Prism version 10 (GraphPad Software, LLC). CD8/Treg ratios are calculated based on actual numbers of these cells collected per sample.

### IFN-γ production by spleen cells *in vitro*

Spleen cells were obtained by mashing spleen pieces between the frosted ends of sterile microscope slides followed by washing through a 70 µm cell strainer. Red blood cells were lysed by hypotonic shock. Spleen cells from naive C57BL/6 mice were cultured at 5 million cells/mL for 3d with Concanavalin A (ConA) at 5ug/mL, then washed 3 times before plating and incubating for another 22-24hrs at 50,000 cells/well with the various concentrations of recombinant mouse IL-12 and recombinant mouse or human IL-2. Plates were spun down and supernatants removed from the cultured splenocytes for dilution and immediate use in ELISA. Mouse interferon-gamma (IFN-γ) ELISA was performed using ELISA MAX Standard Set Mouse IFN-γ (Biolegend, 430801) according to manufacturer’s instructions.

### Statistical analysis

Average group tumor volumes are plotted showing mean +/- SEM. Tumor volume plots were summarized by time-weighted average (area under the volume-time curve, calculated using trapezoidal method). Time-weighted averages were compared between treatment groups overall by Kruskal-Wallis tests. If significant by Kruskal-Wallis test or if only two groups were determined to be compared *a priori*, pairwise comparisons were conducted using Mann- Whitney tests. Survival data were plotted using Kaplan-Meier methods and analyzed using log- rank comparisons. Flow cytometry results are plotted as mean +/- SD, with each symbol representing results from one mouse/sample. Flow cytometry data were analyzed by Student’s T test. Complete response rates were analyzed overall with Chi-Square tests (when comparing three or more groups). If significant by Chi-Square tests or if only two groups were determined to be compared *a priori*, pairwise comparisons were conducted using two-sample tests of proportions. P values < 0.05 were considered significant. The experiments were repeated twice unless stated otherwise. Statistical analyses were done either on a single representative experiment or on pooled (cumulative) experiments as specified in the Figure legends.

## Results

### Effect of CRI against late stage B78 tumors

**Figure 1:**
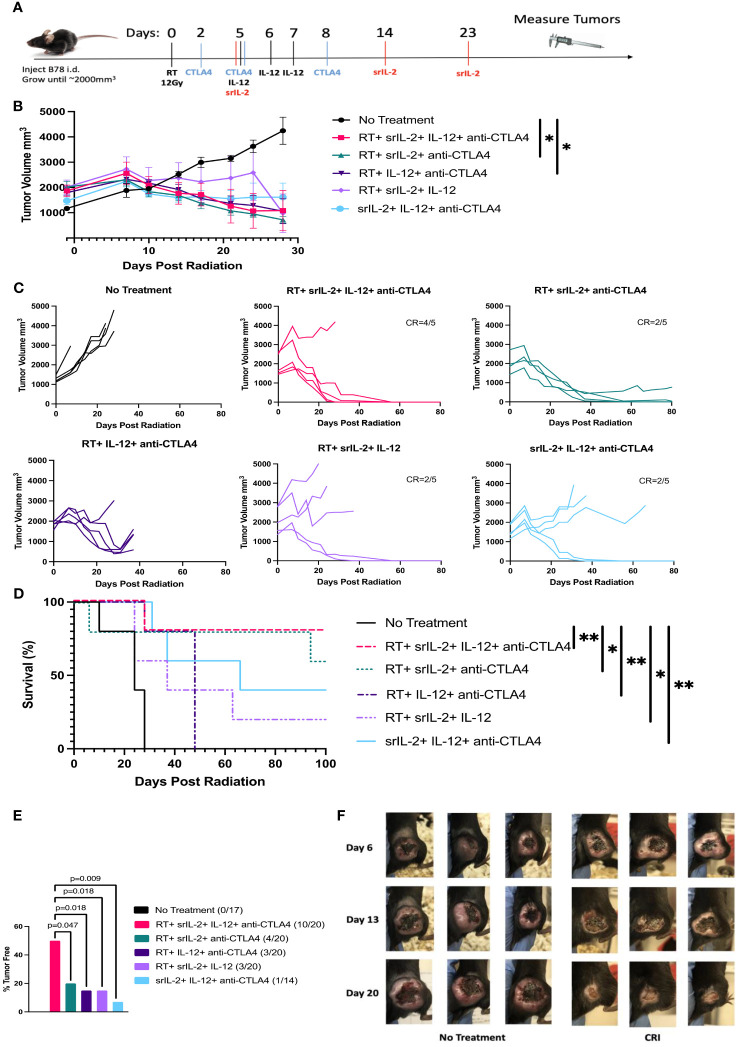
CRI elicits a robust antitumor response against advanced B78 melanoma tumors and extends survival. **(A)** C57BL/6 mice were injected i.d. with 2x10^6^ B78 cells on their right flank. Treatment began when tumors reached a volume of 1500-2000mm^3^. On day 0, mice received 12 Gy radiation to their right flank. On days 2, 5, and 8, mice were injected with anti-CTLA-4 mAb i.p. On days 5, 6, and 7 IL-12 was administered i.t. On days 5, 14, and 23, srIL-2 was administered i.v. Control mice received no treatment. Tumor volume was measured with calipers. **(B)** Mean +/- SEM of tumor volumes calculated for the mice that remain alive at the indicated time points. The data from a representative experiment of 3 similar experiments are shown. **(C)** Individual mouse tumor growth curves from the same representative experiment as **(B)** are shown. In all groups, the tumor volume line is stopped at the time point when that individual mouse had to be euthanized according to the animal protocol guidelines regarding tumor size and/or symptoms. The number of mice becoming and remaining tumor-free for 100 days (complete response =CR) out of 5 mice is indicated for groups with mice with sustained CR. **(D)** Survival rates of treated mice for those shown in **(B, C, E)** Combined sustained CR rates (% tumor free), determined at day 100 following RT, from four separate similar experiments were compiled. **(F)** Representative photos of tumors of three mice in the no treatment group and three mice in the CRI group at days 6, 13, and 20 following treatment start. Significance, as shown on the graphs, corresponds to the following p-values, *p<0.05, **p<0.01.

We recently published that a combination of one fraction of 12 Gy RT, srIL-2 and anti-CTLA-4 could induce complete regression of large B78 tumors with tumor volume of 1000 mm^3^ in about 50% of mice (10). We hypothesized that this therapy could result in regression of even larger tumors when supplemented with i.t. IL-12, a cytokine that is potent in inducing tumor regression when delivered locally (20–22). In addition, IL-12 has been shown to synergize with IL-2 to induce antitumor effects (11–13). We confirmed the previously demonstrated (23) *in vitro* synergy of IL-2 (both mouse and human) with mouse IL-12 on Concanavalin A (Con A) - activated mouse spleen cells by measuring production of IFN-γ (**Supplementary Figure 1**). To test the antitumor effect of CRI, C57BL/6 mice were injected with B78 melanoma cells i.d. into the right flank. When the tumors achieved a volume of ~1200-3000 mm^3^, mice were randomized, assigned to treatment groups with a mean volume of 1500-2000 mm^3^ and given therapy starting with the RT. The day RT was given is designated as day 0 (**Figure 1A**). The six treatment groups included treatment with all four components of CRI (RT, srIL-2, IL-12, anti-CTLA-4), the three possible combinations of three CRI components, and no treatment (control). The full CRI regimen combining all four components, RT, srIL-2, IL-12, and anti-CTLA-4 induced rapid (within 3-4 weeks) complete regression of most tumors (4 of 5) in this group; the only tumor that did not regress was one that had a size of ~2600 mm^3^ when the treatment started, although a separate tumor of a similar size still responded to that treatment (**Figures 1B, C**). The 4 full-CRI treated mice whose tumors were eradicated remained tumor-free (**Figure 1D**). The combined data of four similar experiments showed that the CRI regimen of all four components induced a significantly better antitumor response than any combination of three components, documenting that each of the four components contributed to the full effect of the full CRI regimen (**Figure 1E**). Representative photographs of control and CRI-treated mice during and after treatment are presented in **Figure 1F**.

### Systemic effect of CRI in the B78 model

**Figure 2:**
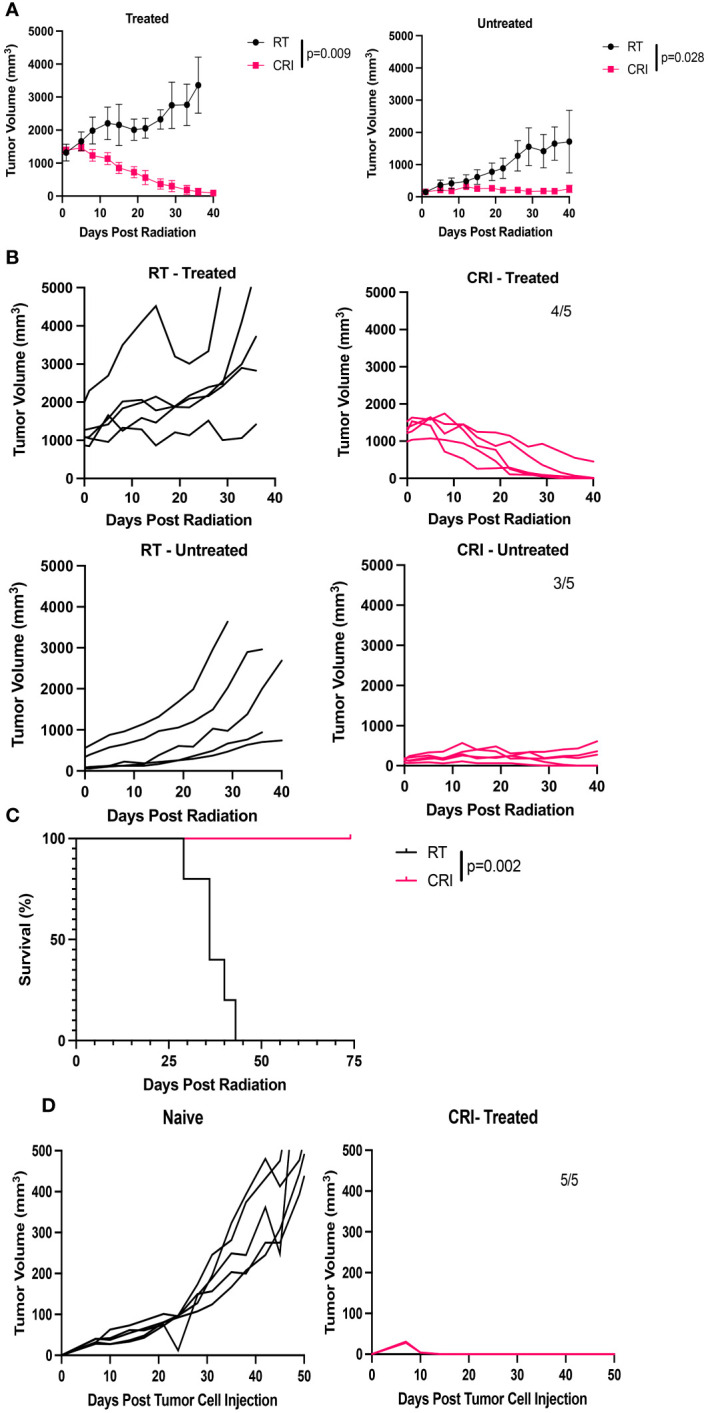
CRI induces a systemic antitumor effect and immune memory in mice bearing B78 melanomas. **(A)** Mean +/- SEM of tumor volumes for mice bearing a ~10 week established tumor in the right flank, and a 19 day established tumor in the left flank. Mice were then randomized: the CRI group received RT + i.t. IL-12 to only the larger primary (right) tumor with no RT or i.t. IL12 to the smaller (left) tumor together with systemic srIL-2 and anti-CTLA-4. Control mice received RT of the tumors at the right flank. “Treated” – primary (right) tumors; “untreated” – secondary (left) tumors. **(B)** Individual mouse tumor curves following treatment for the mice shown in **(A)**. Numbers of mice which became and remained tumor-free out of total number of mice are shown. **(C)** Mouse survival following treatment for the mice shown in **(A, B)**. **(D)** Tumor curves following B78 tumor rechallenge with 2x10^6^ B78 tumor cells injected into the 5 CRI- treated mice that rejected both of their tumors in **(A–C)**, compared to growth of 2x10^6^ B78 tumor cells in naïve mice. p-values are shown for statistically significant comparisons. The representative data from one of two experiments are shown.

We hypothesized that CRI could induce systemic antitumor effects against established distant tumors. To test this hypothesis, C57BL/6 mice were injected with 2x10^6^ B78 tumor cells in the right flank (primary tumor) and seven weeks later were injected with 2x10^6^ B78 tumor cells in the left flank. Nineteen days after the second tumor inoculation, mice were randomized into 2 groups; one group received CRI (with RT and IL-12 administered to the tumors on the right flank, srIL-2 and IL-12 administered systemically), while the other group received RT alone to the tumors on the right flank only. Two similar independent experiments were performed. At the beginning of CRI, the mean primary tumor volume was 1,380 mm^3^ in one experiment and 1,196 mm^3^ in the second experiment. The mean volume of tumors in the left flank was 129 mm^3^ in one experiment and 20 mm^3^ in the second experiment. The results of the first mentioned experiment are shown in **Figure 2**. CRI induced statistically significant regression of the tumors on both flanks, with 4 of 5 mice rejecting treated tumors and 3 of 5 mice rejecting distant tumors (**Figures 2A, B**), and resulted in increased survival (**Figure 2C**). In the 2^nd^ experiment, 3 of 5 mice rejected treated tumors and 3 of 3 mice rejected distant tumors (2/5 mice in that experiment did not have secondary tumors present at the start of treatment). When data from both experiments are combined, CRI induced complete tumor regression of primary tumors in 9/10 mice, and secondary tumors in 6/8 mice. These tumor-free mice developed immunological memory, as they rejected rechallenge with B78 tumor cells (**Figure 2D**).

We hypothesized that the systemic effect of CRI involved an *in situ* vaccine effect, resulting in part from the local administration of RT and i.t. IL-12 acting on the primary tumor, when combined with the systemic srIL-2 and anti-CTLA-4. To test this hypothesis, mice bearing a single B78 tumor, on the left flank, were treated with RT administration and IL-12 injection to the skin of the right flank (in the absence of any tumor), together with systemic injections of srIL-2 and anti-CTLA-4. The results in **Supplementary Figure 2** show that this treatment eradicated tumors in 2 of 5 mice with a mean volume 37 mm^3^ (**Supplementary Figure 2B**) but did not induce complete tumor regression when the tumors had a mean volume 62 mm^3^ (**Supplementary Figure 2C**). In contrast, in CRI-treated mice where RT and IL-12 were applied to the primary tumors in a two- tumor model, 3/5 mice eradicated distant tumors with a mean tumor volume 129 mm^3^ (**Figure 2B**). These results suggest that CRI with local RT and i.t. IL-12 induces a stronger systemic antitumor effect, at least in part, via the local effect of the RT and i.t. IL-12 acting on the right flank tumor, potentially via an *in situ* vaccine effect.

### Role of T cells in the antitumor effects of CRI against B78 melanomas

**Figure 3:**
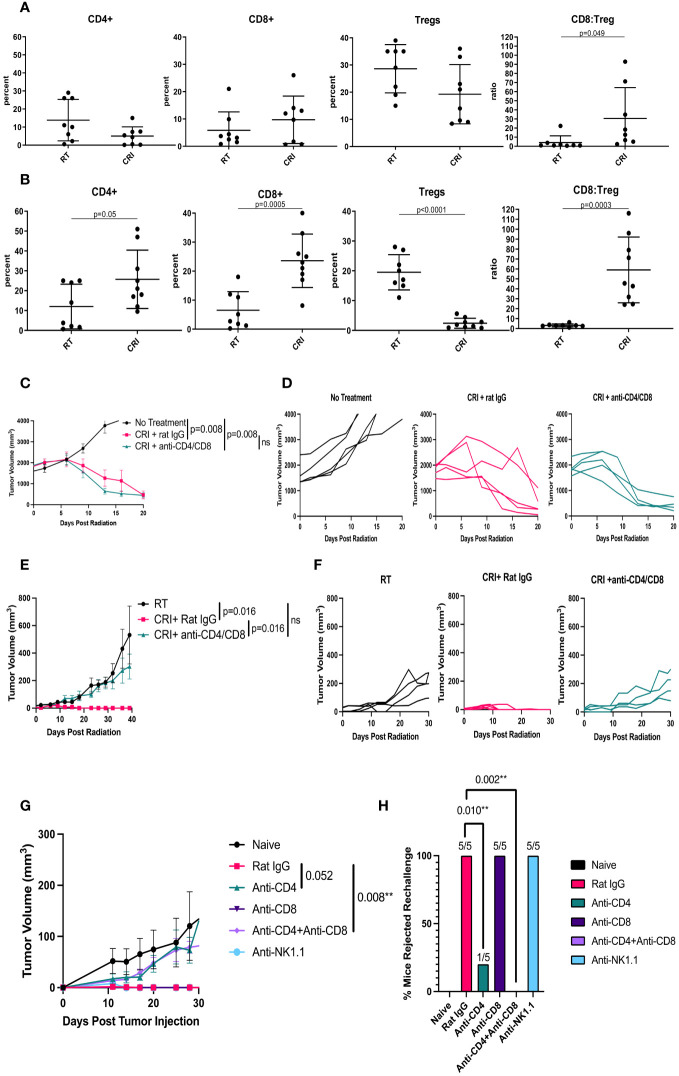
Role of T cells in CRI-induced antitumor effect and immune memory. **(A, B)** – Flow cytometry of TME. C57BL/6 mice bearing advanced B78 tumors were treated with RT or CRI as described in the **Figure 1** legend. On day 8 **(A)** or 12 **(B)** tumors were collected, digested, and single cells were analyzed by flow cytometry. The cells were gated on live single CD45^+^ cells. Mean +/- SD and individual values of percentages of CD4^+^, CD8+ T cells and Tregs (expressed as a % of CD4 cells), and ratios of CD8^+^ T cells: Tregs are shown. The combined data from two similar experiments are shown. **(C-H)**: Role of T cells and NK cells in CRI effect. **(C)** Mean +/- SEM of tumor volumes and **(D)** individual mouse tumor curves in mice bearing a single B78 tumor are shown following CD4 and CD8 T cell depletion during CRI treatment. The representative data from one of two experiments are shown. **(E)** Mean +/- SEM of tumor volumes for the left flank tumor and **(F)** individual tumor curves for the left flank tumor, in mice bearing two B78 tumors (a large tumor on the right and a small tumor on the left as in **Figure 2**) are shown following CRI treatment with CD4 and CD8 cell depletion or with control rat IgG. The representative data from one of two experiments are shown. **(G)** Mean +/- SEM of tumor volumes following CD4, CD8, or NK cell depletion during B78 tumor rechallenge in mice that rejected large B78 tumors. As all 5 mice in each group receiving rat IgG (red), anti-NK1.1 (turquoise) and anti-CD8 (purple) rejected the rechallenge tumors, all 3 of these lines are superimposed on each other along the X- axis, and difficult to see. **(H)** Percentages of mice that rejected rechallenge, calculated from the number of tumor-free mice out of the total number of mice (n=5) in each group are shown. A representative of two experiments is shown with p-values.

We investigated the effect of CRI vs. RT alone on immune cells within B78 tumor microenvironment (TME) on days 8 and 12 by flow cytometry. Day 8 was selected as the time point at the onset of tumor regression, and day 12 as the time point in the process of tumor regression. The results in **Figure 3A** show that on day 8, there was a significant (p=0.049) increase of CD8/Treg ratio between the CRI and control group. On day 12, the effects of CRI on immune infiltrate were more pronounced, inducing a reduction of the percentage of Tregs (p<0.0001), an increase of the CD8/Treg ratio (p=0.0003), and increases in the CD4 (p= 0.05) and CD8 (p = 0.0005) infiltration (**Figure 3B**). Percentages of other cells such as F4/80^+^ macrophages and CD11b^+^Gr-1^+^ myeloid-derived suppressor cells were not reproducibly affected (data not shown). The means +/- SD are presented in **Supplementary Table 1**.

The observed changes in T cells in the TME during tumor regression, the regression of distant tumors in mice with 2 tumors, where only one tumor received RT and i.t. IL-12 (**Figures 2A-C**), and the development of immunological memory in tumor-free mice (**Figure 2D**) all suggested that B78 tumor regression following CRI was mediated, at least in part, by T cells. To test the role of T cells in the observed antitumor effects, mice bearing large B78 tumors were depleted of T cells with anti-CD4 plus anti-CD8 antibodies before and during CRI treatment. The efficacy of CD4 and CD8 depletion in the tumors was confirmed in a parallel experiment (**Supplementary Figure 3**). The results in **Figures 3C, D** show that in mice bearing a single B78 tumor, depletion of T cells did not affect CRI-induced rapid tumor regression during the first three weeks. Similarly, depletion of NK cells did not affect CRI-induced tumor regression (**Supplementary Figure 4**), suggesting that cells other than CD4 or CD8 T cells, or NK cells, were responsible for this early regression of large tumors. In contrast, in the two-tumor B78 model shown in **Figure 2**, where local RT and i.t. IL-12 to the right flank combined with systemic srIL- 2 and anti-CTLA-4 enabled eradication of both tumors in many mice (**Figure 2C**), a similar depletion of CD4 and CD8 T cells significantly reduced the antitumor effect against the secondary tumor in the left flank (**Figures 3E, F**). This would be consistent with CRI inducing a potent, non-T cell mediated, response against the large tumor in the right flank, and in the process inducing a systemic adaptive T cell response able to subsequently act on inhibiting the growth of the smaller distant tumor. We also tested the role of T cells in memory immune responses. Mice that rejected large B78 tumors following CRI were rechallenged with B78 tumor cells and received either anti-CD4 mAb, anti-CD8 mAb, a mixture of anti-CD4/anti-CD8 mAb, anti-NK1.1 mAb or control rat IgG. Naïve mice were injected with B78 cells as a control. The results in **Figures 3G, H** show that T cells, notably CD4^+^ T cells, were responsible for the antitumor effect against the tumor rechallenge.

### Effect of CRI against MC38 adenocarcinoma

**Figure 4:**
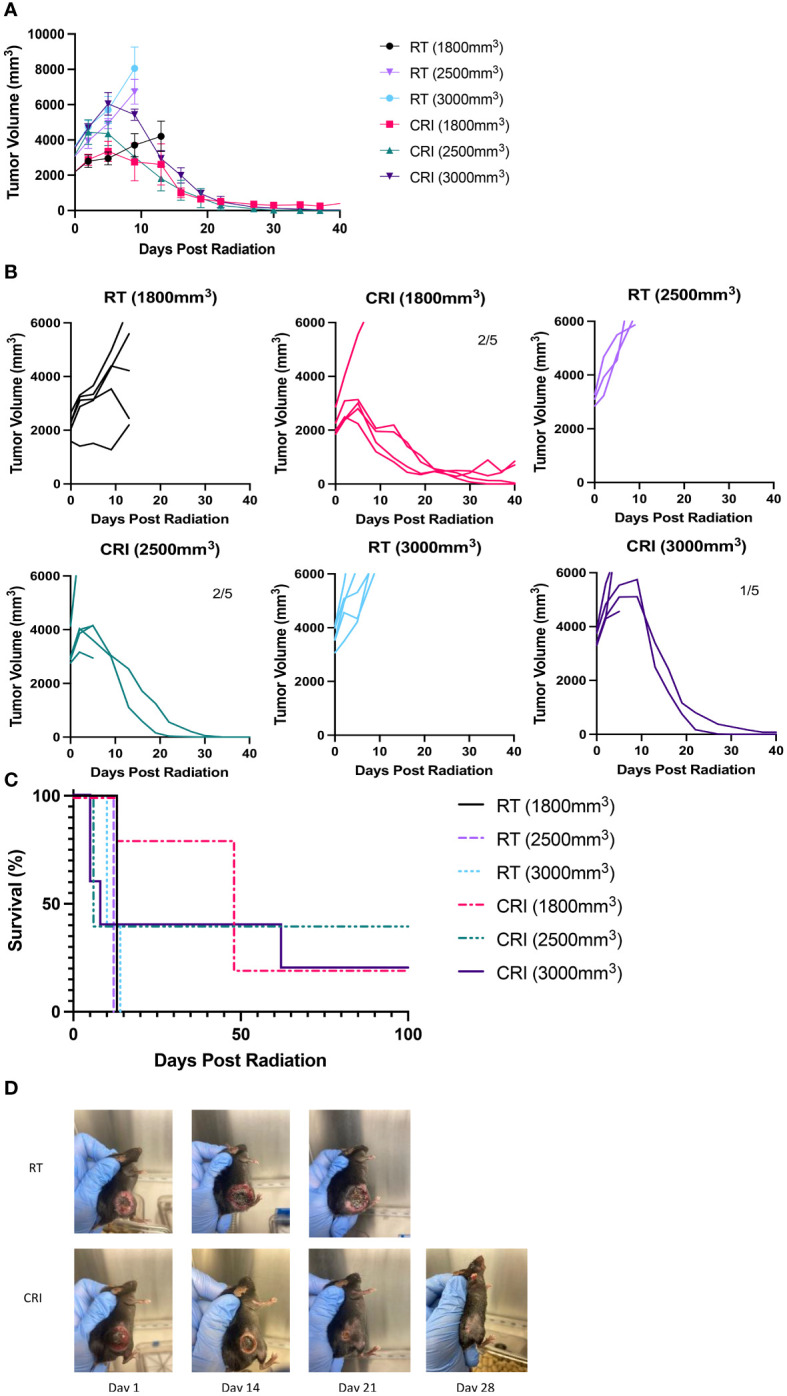
CRI induces regression of late-stage MC38 tumors and extends survival. **(A)** Mean +/- SEM of tumor volumes and **(B)** individual mouse curves following CRI treatment or RT are shown for separate groups of mice with starting tumors of 1800, 2500 or 3000 mm^3^. Numbers on graphs indicate numbers of tumor-free mice out of total mice as of day 44 following radiation. Numbers in parenthesis indicate average tumor volumes at the beginning of treatment. In the last graph (CRI, 3,000 mm^3^), two mice showed rapid tumor regression; one mouse became tumor-free and another one did not. **(C)** Mouse survival following treatment. **(A–C)** show the representative data from one of two experiments. **(D)** Serial pictures of MC38 tumors on days 1, 14, 21, and 28 following RT or CRI, for a single representative mouse from each group involved in a separate experiment than the others pictured in **(A–C)**. On day 1, the tumor volume for the RT mouse was 2851 mm^3^ and for CRI mouse 2458 mm^3^. A photo of the RT-treated mouse is not provided for day 28 because it was euthanized due to the large tumor. The results of a representative experiment (separate from other experiments shown in **(A-C)** are shown.

Next, we asked if the striking and rapid regression of late-stage B78 melanoma could be reproduced in other mouse tumor models. We tested the effect of CRI against a fast-growing MC38 adenocarcinoma in syngeneic C57BL/6 mice at late stages of tumor growth. Different groups of mice bearing a single flank tumor, with mean tumor volumes at the start of treatment averaging 1800, 2500 and 3000 mm^3^, received CRI vs. RT alone (**Figures 4A, B**). The results show that CRI induced rapid regression of tumors in each treatment group, even tumors as large as 3000 mm^3^ (~18 mm in diameter). CRI resulted in extended survival with complete tumor regression in 20-40% of mice (**Figure 4C**). The overall statistical analysis of all three CRI- and three RT-treated groups showed that on day 27, 13/13 mice were dead in all RT-treated groups and only 7/15 mice were dead in all CRI-treated groups (p = 0.002, Chi-Square test). Pictures of a representative RT-treated and a CRI-treated mouse are shown in **Figure 4D**.

### Changes in TME following CRI of advanced MC38 tumors

**Figure 5:**
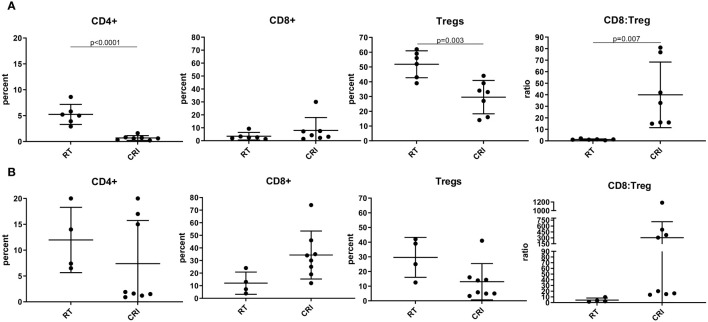
Effect of CRI treatment on T cells in MC38 TME. C57BL/6 mice bearing advanced MC38 tumors were treated with RT (control) or CRI. On day 8 **(A)** or 12 **(B)** tumors were collected, digested, and single cells were analyzed by flow cytometry. The cells were gated on live single CD45^+^ cells. Mean +/- SD and individual values of percentages of CD4^+^, CD8+ T cells (expressed as a % of the CD45 cells) and Tregs (expressed as a % of the CD4 cells), and ratios of CD8^+^ T cells: Tregs are shown. The combined data from two similar experiments are shown.

Flow analysis of CRI-treated MC38 TME showed, in agreement with the results obtained in the B78 tumor model (**Figures 3A, B**), the reduction of Tregs and increase of the CD8/Treg ratio (**Figure 5**). However, in contrast with the B78 model where significant differences were more pronounced on day 12 vs. day 8, in the MC38 model the effects of CRI on T cell populations were more pronounced on day 8 vs. day 12 (although the lack of statistical significance of the CD8/Treg ratio on day 12 is probably due to the high variability in CRI-treated mice). This likely reflects the more immunogenic nature of MC38 tumors which may well enable an earlier onset of the immune response. The means +/- SD of these flow experiments are presented in **Supplementary Table 1**.

### Role of T cells in the antitumor effects of CRI against MC38 adenocarcinomas

**Figure 6:**
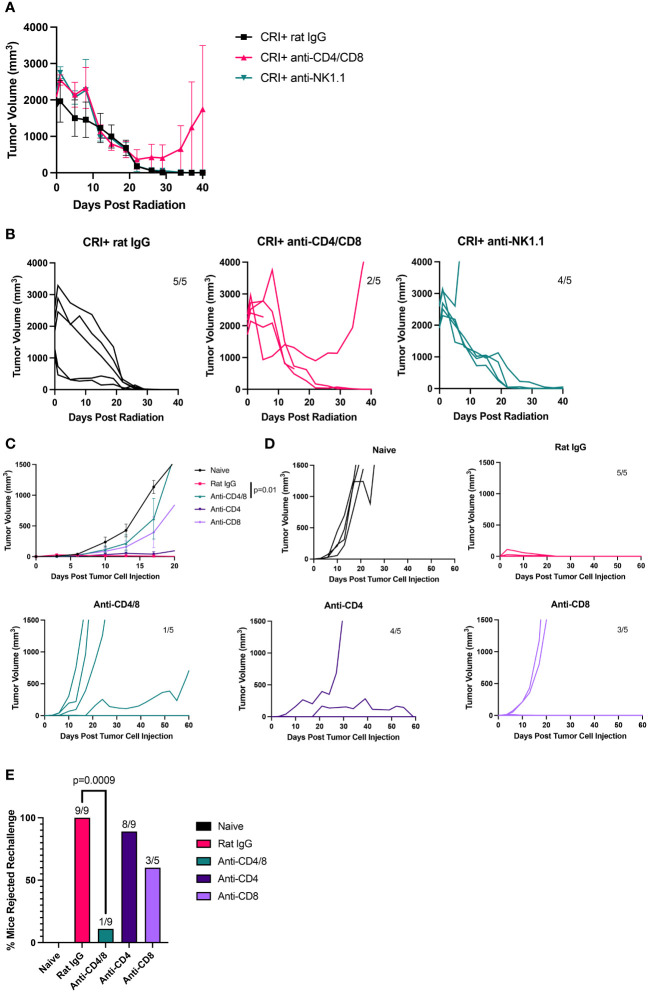
CD4 and CD8 T cells are not required for CRI treatment-induced rapid regression of large MC38 tumor rejection but are necessary for rejection of tumor rechallenge. **(A)** Mean +/- SEM of tumor volumes and **(B)** individual mouse tumor curves are shown following treatment with CRI and either rat IgG, anti-CD4 +anti-CD8, or anti-NK1.1 antibodies. The data of a representative experiment from 3 experiments are shown for T cell depletion (except for the NK cell depletion, which was performed in only one experiment). **(C)** Mean +/- SEM of tumor volumes and **(D)** individual mouse tumor curves following rechallenge with MC38 cells. Naïve mice (5) and 20 mice previously cured of MC38 tumors by the CRI regimen were divided into groups of 5 mice. All were implanted with MC38 tumor cells and different groups of the previously cured mice received either Rat IgG, anti-CD4/8, anti-CD4 or anti-CD8 depleting antibody. Numbers on graphs indicate the number of tumor-free mice out of total mice. The representative data from one of two experiments are shown. **(E)** The bar graph shows percentages of tumor-free mice compiled from two experiments (except the anti-CD8-treated group was tested in only one experiment) following MC38 tumor rechallenge of mice that rejected M38 tumors following CRI treatment.

Next, we tested the role of T cells and NK cells in CRI-induced tumor regression in the MC38 tumor model. As in the B78 tumor model (**Figure 3A**; **Supplementary Figure 4**), this tumor regression could be observed in mice depleted of T cells and NK cells (**Figures 6A, B**) and resulted in immunological memory (**Figures 6C, D**). As shown for the B78 model (**Figures 3G, H**), immune memory in cured mice was dependent on T cells (**Figures 6C-E**). However, whereas CD4 T cells played a major role in the B78 model, only depletion of both CD4 and CD8 cells significantly prevented the rejection of MC38 rechallenge by previously cured mice.

### Effect of CRI against CT26 adenocarcinoma and 9464D neuroblastoma

**Figure 7:**
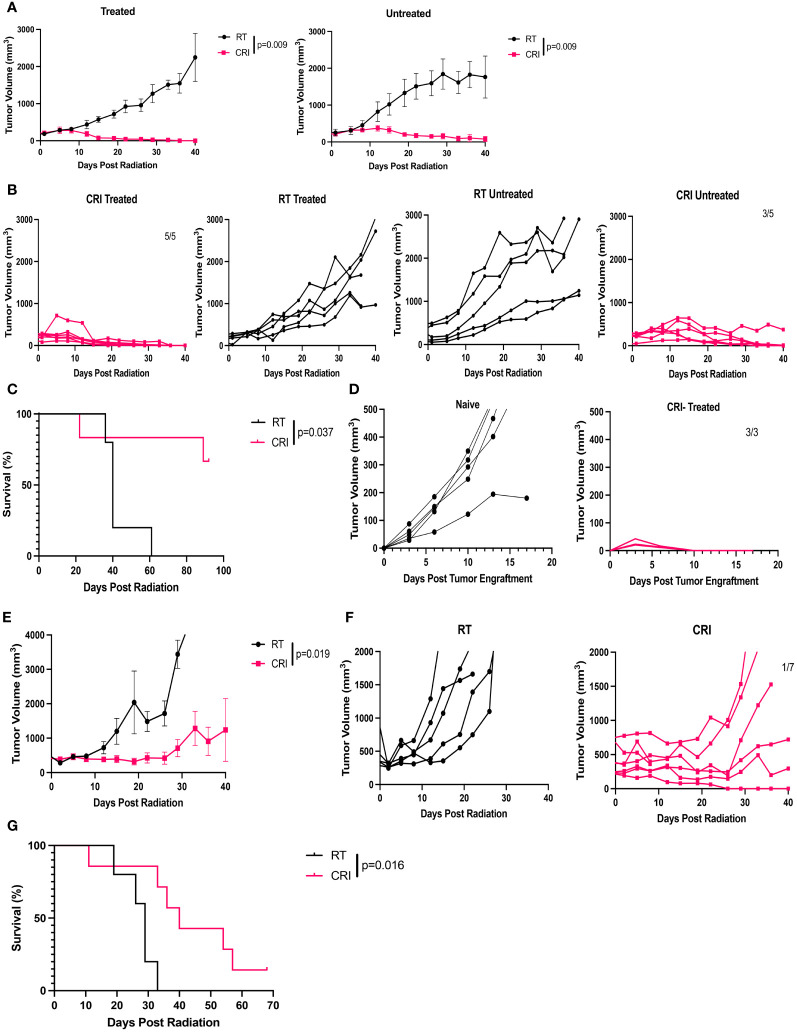
Antitumor effects of CRI in two-tumor CT26 colon carcinoma model and in 9464D-GD2 neuroblastoma model. **(A–C)** Mice bearing similar sized bilateral flank CT26 tumors (implanted in the right and left flanks on the same day) received RT alone, with the RT delivered only to the right flank “treated” tumor, or CRI, including RT and i.t. IL-12 to the right flank “treated” tumor. At the time treatment was started the mean tumor volume on the right side was 197 mm^3^ and on the left side 261 mm^3^. **(A)** Mean +/- SEM of tumor volumes for the treated and untreated tumors. **(B)** tumor growth curves for individual mice for untreated and treated tumors, and **(C)** survival are shown. **(D)** Individual mouse tumor curves for 5 naïve mice and 3 mice from **(A–C)** that remained tumor-free following initial treatment and subsequent re-implantation of CT26 cells. **(E–G)** Mice bearing a single 9464D-GD2 neuroblastoma in the R flank (starting tumor size ~500 mm^3^) received RT alone, or CRI. **(E)** Mean +/- SEM of tumor volumes, **(F)** individual mouse curves, and **(G)** survival of mice bearing a single 9464D-GD2 tumor receiving RT or CRI. The ratios shown on the tumor growth graphs in **(B–F)** indicate the number of tumor-free mice out of total mice per group. P-values are shown for statistically significant differences. The data shown here for mice bearing either CT26 or 9464D-GD2 tumors are representative of two separate similar studies in each model.

In addition to B78 melanoma and MC38 adenocarcinoma, we tested the effect of CRI against CT26 colon adenocarcinoma in a two-tumor model. In this model, CT26 tumor cells were injected into syngeneic Balb/c mice in the right and left flanks on the same day, and RT was given when the tumor volumes reached 190-260 mm^3^. We used these smaller tumor sizes because CT26 tumors grow rapidly and ulcerate quickly at larger tumor volumes; this ulceration necessitates mouse euthanasia before the larger tumors can be adequately treated or evaluated for tumor regression. The results show that CRI induced suppression of tumor growth and regression of both tumors; both the one treated locally with RT and i.t. IL-12 on the right side and the tumor on the left side that did not receive RT and i.t. IL-12 (**Figures 7A–C**). The mice that rejected both CT26 tumors developed immunological memory as they rejected rechallenge with CT26 cells (**Figure 7D**). Similar to our findings in the B78 tumor model (**Supplementary Figure 2**), the results in **Supplementary Figure 5** show that CRI, in mice with a single tumor, where RT and IL-12 were given to normal skin on the flank opposite the CT26 growing tumor, slowed tumor growth but did not induce complete tumor regression when the tumors had a mean volume of 210 mm^3^. In contrast, RT and IL-12 applied to the primary tumors in combination with systemic srIL-2 and anti-CTLA-4 in a two-tumor model, induced eradication of 5 out of 5 of the primary tumors (mean volume 197 mm^3^), and 3 out of 5 of the distant tumors (mean volume 261mm^3^) (**Figure 7B**). These results are consistent with the findings in the B78 tumor model that CRI with the local RT and the i.t. IL-12 given to the tumor directly induces a systemic antitumor effect, at least in part, via an *in situ* vaccine effect.

9464D-GD2 is a “cold” neuroblastoma model with low mutational burden (15) and low MHC class I expression (24); mice with a small neuroblastoma (75 mm^3^ or less) show no response to a combination of RT + anti-CTLA-4 + anti GD2/IL-2 immunocytokine, and ~ 50% of mice bearing these small 9464D-GD2 tumors regress in response to a combined regimen using RT, immunocytokine, anti-CD40, CpG and anti-CTLA-4 (15, 24). However, mice with 9464D-GD2 tumors do not regress in response to this same regimen when tumors are > 150mm^3^ (unpublished data from our lab). Even so, CRI treatment of mice bearing single 9464D-GD2 tumors with a mean diameter of ~500 mm^3^ resulted in statistically significant suppression of tumor growth and extended survival with one CRI-treated mouse rejecting the tumor (**Figures 7E–G**).

## Discussion

Numerous experimental studies in tumor-bearing mice have shown that a variety of immunotherapy regimens can be effective against syngeneic and spontaneous tumors. In most of these studies the tumors were relatively small at the time treatment was started, and the efficacy of the immunotherapy was inversely correlated with the size of the tumor as previously shown by us (25, 26) and others (7). Large tumors usually need to be debulked either surgically (4, 5, 27) or by chemotherapy (6) to facilitate immunotherapy-mediated antitumor responses. In this report we show that even without tumor debulking, CRI (a combination of RT, srIL-2, IL-12 and anti- CTLA-4), can induce complete regressions of some late-stage tumors with tumor volumes of 2000 mm^3^ or larger. The rationale for selecting the components of the CRI regimen is based on different and complementary mechanisms of their action: local RT induces tumor cell death and tumor antigen release thus serving as *in situ* vaccine (19); srIL-2 induces T/NK cell activation, proliferation and survival (18); anti-CTLA-4 induces T cell activation via checkpoint blockade (1–3) and Treg depletion via antibody-dependent cell-mediated cytotoxicity through an Fc effector mechanism (17, 28, 29); and IL-12 induces T/NK cell activation and IFN-γ release leading to macrophage activation (22). Future optimization of this regimen, e.g., minimizing potential toxic effects of IL-12 by using a fusion protein or slow release form (22), and/or using different checkpoint inhibitors, is warranted for clinical testing.

In the weakly-immunogenic B78 melanoma model, as well as in the immunogenic MC38 colon carcinoma model, CRI induced rapid regression (within 3 weeks) of very large late-stage tumors. T cells did not play a critical role in the initial regression of large single B78 tumors treated with CRI (**Figures 3C, D**). In contrast, the antitumor effect on a secondary distant smaller B78 tumor was reduced in T cell-depleted mice (**Figures 3E, F**). These results are in agreement with our previous data showing that the initial suppression of growth of much smaller B78 tumors in response to RT, srIL-2 and anti-CTLA-4 was also observed in T cell-depleted mice, whereas the therapeutic effect against lung metastases was reduced in those same T cell depleted mice (10). These results suggest that the rapid initial regression (within three weeks of CRI treatment) of large tumors, which received local RT and IL-12 treatments, is not mediated by T cells. However, this local RT + IL-12 therapy at the large right flank tumor site in combination with systemic srIL-2 and anti- CTLA-4 induced a systemic antitumor effect mediating an adaptive, T cell dependent, response at the distant left tumor site that did not get direct RT + IL-12 (**Figure 3E**). This systemic immune response affecting the left flank tumor was stronger than when mice with a small left flank tumor received systemic srIL-2 and anti-CTLA-4, together with RT and subcutaneous IL-12 given to the skin on the distant right flank, in mice not bearing a tumor in the right flank (**Supplementary Figure 2C**). These findings suggest that RT and IL-12 given locally to an existing large tumor, together with systemic srIL-2 and anti-CTLA-4 induced an *in situ* vaccine effect.

We also noted an increase in CD4 T cells seen 12 days after CRI for B78 tumors but not for MC38 tumors (**Figures 3**, **5**). CD4 T cells played a major role in the memory (but not the primary) response against B78 tumors (**Figure 3H**), whereas CD8 T cells played a major role in memory response against MC38 tumors (**Figure 6E**). These differences may relate to a separate role for CD4 cells in response to B78 tumors than MC38 tumors, in part due to the lack of MHC class I and presence of MHC class II expression on B78 cells, which favors CD4 T cell response, and high MHC class I expression on MC38 cells favoring CD8-mediated T cell response. These issues related to the role of CD4 cells in B78 responses are the focus of separate studies beyond the scope of this manuscript (Erbe A. et al, in preparation).

Similar to depletion of T cells, depletion of NK cells did not interfere with the rapid regression of B78 tumors, suggesting that NK cells do not play a major role. It remains unclear which cells mediate rapid regression of large tumors within three weeks after CRI is started. As neither T nor NK cells are involved in this initial anti-tumor effect, the remaining likely candidates are macrophages (30), neutrophils (31), or other myeloid cells (32). We hypothesize that rapid tumor regression following CRI is mediated by macrophages. Because large tumors become necrotic as seen in **Figures 1F** and **4D**, macrophages in TME are likely to phagocytose necrotic tumor tissues, and additional activation by IL-2 and IL-12 can cause these macrophages to mediate tumor regression, as was reported (30).

In several cases, the noted variability of tumor measurements is caused by ulceration and/or necrosis of the very large tumors. There was also variability in the number of complete responses to the treatments in individual experiments. However, despite this variability, CRI reproducibly showed an overall better level of antitumor efficacy.

CRI was able to induce complete tumor regression for at least some treated mice in each of the four tumor models we tested: B78 melanoma, MC38 adenocarcinoma, CT26 adenocarcinoma and 9464D-GD2 neuroblastoma. These results suggest that this combined treatment has a wide applicability among various murine tumors. However, whereas the majority of mice bearing B78, MC38 or CT26 tumors showed complete tumor regression without ever showing subsequent tumor regrowth following CRI, the majority of mice bearing 9464D-GD2 neuroblastomas exhibited suppression of tumor growth without eradication of growing tumor.

These results are in agreement with our previous studies showing that 9464D-GD2 tumors were much less responsive than B78 tumors to a separate combination of RT and immunotherapy (15, 24). The mechanisms of this relative resistance of 9464D-GD2 tumors are unknown and may be due to the features of tumor cells or TME. Since the addition of macrophage activators anti- CD40 and CpG improved the antitumor efficacy of RT and 14.18-IL2 immunocytokine in this “cold” tumor model (15, 23, 24) and in the B78 tumor model (33), it is possible that this CRI regimen may be improved for the 9464D-GD2 model by the addition of these or similar activators of innate immunity. Additional mechanistic studies, beyond the scope of this manuscript, will be needed to clarify more precisely how the addition of IL-12 enables the more potent efficacy of this regimen for tumors like B78, CT26 and MC38, and why the much colder 9464D-GD2 tumor is responsive, but less so, to this regimen.

These studies are clinically relevant, because checkpoint blockade immunotherapy, either alone or in combination with RT, can result in regression for some patients with certain solid tumors, particularly those with higher immunogenicity and high mutation burden (2, 34). This includes some, but not the majority of, patients with melanoma, whereas it is less effective in most patients with low immunogenic tumors which have a low tumor mutation burden, such as many patients with melanoma and nearly all patients with pediatric cancers such as neuroblastoma (35, 36). Our results show that the addition of IL-2 and IL-12 enables more potent responses to RT and checkpoint blockade for moderately immunogenic MC38 and CT26 colon adenocarcinomas, relatively cold B78 melanoma, and very cold 9464D-GD2 neuroblastoma. Even so, the response to this CRI regimen is more complete for the MC38, CT26 and B78 melanoma than for the 9464D-GD2 neuroblastoma. We are not implying that this regimen is yet ready to apply to all melanomas, colon adenocarcinomas or neuroblastomas; rather, our data suggest that this more complex combination may offer potentially better responses for tumors insufficiently immunogenic to respond to checkpoint blockade with or without RT.

Overall, our results indicate that this CRI regimen has significant anti-tumor activity against a variety of advanced large murine tumors with different histology, strain of origin and immunogenicity. Further development is underway to determine what additional modifications in this regimen may augment the strong antitumor efficacy demonstrated. Additional work with future attention to safety considerations is needed to evaluate the modifications required to translate this approach into future clinical testing.

## Data availability statement

The original contributions presented in the study are included in the article/**Supplementary Material**. Further inquiries can be directed to the corresponding author.

## Ethics statement

The animal study was approved by UW Madison School of Medicine and Public Health Institutional Animal Care and Use Committee. The study was conducted in accordance with the local legislation and institutional requirements.

## Author contributions

AR: Conceptualization, Investigation, Supervision, Writing – original draft, Writing – review & editing. NT: Data curation, Formal analysis, Investigation, Writing – review & editing. MF: Data curation, Investigation, Writing – review & editing. JZ: Formal analysis, Software, Writing – review & editing. SM: Data curation, Investigation, Writing – review & editing. AE: Investigation, Writing – review & editing. AP: Investigation, Writing – review & editing. DS: Investigation, Writing – review & editing. EC: Investigation, Writing – review & editing. CW: Investigation, Writing – review & editing. WO: Conceptualization, Methodology, Writing – review & editing. ZM: Conceptualization, Funding acquisition, Writing – review & editing. PS: Conceptualization, Funding acquisition, Project administration, Supervision, Writing – review & editing.
