# Supplementary material for: A combined radio-immunotherapy regimen eradicates late-stage tumors in mice
Source: Front Immunol. 2024 Jul 15;15:1419773. doi: 10.3389/fimmu.2024.1419773 (PMC11284032; doi:10.3389/fimmu.2024.1419773)
Supplement: Supplementary file 1 [file DataSheet_1.pdf]

Supplemental Material.

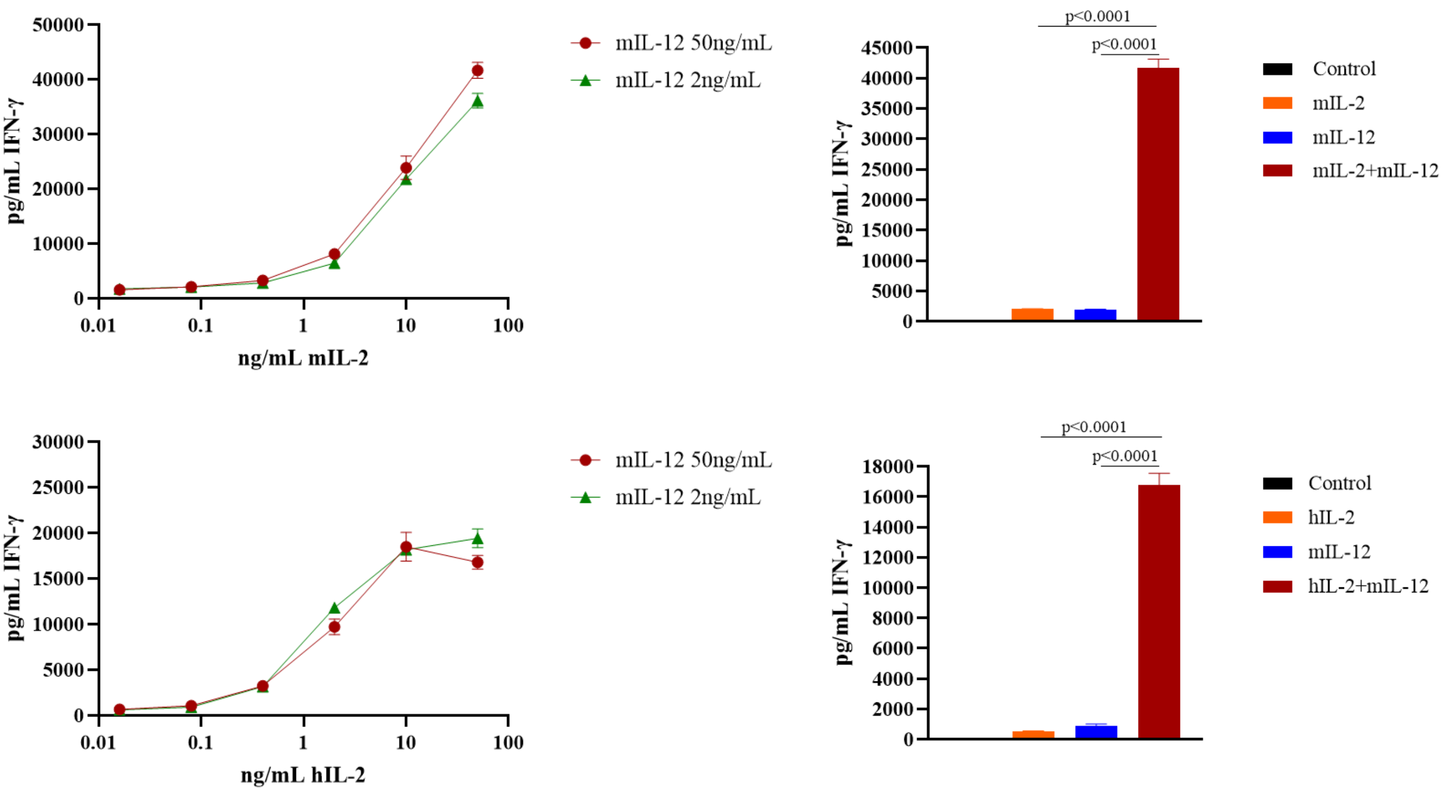

Supplemental Figure 1. Synergy of IL-2 and IL-12 in vitro. C57BL/6 spleen cells were activated with 5 mcg/ml of ConA for 3 days. ConA blasts were washed and incubated with either 2 or 50 ng/ml of recombinant mouse IL-12 and either mouse (A) or human (B) IL-2 for 23 hours. Supernatants were collected and tested for mouse IFN-γ using ELISA. Bar graphs on the right show IFN-γ levels for supernatants from cells treated with IL-12, or IL-2, or their combination, at doses of 50 ng/ml for each of the indicated agents.

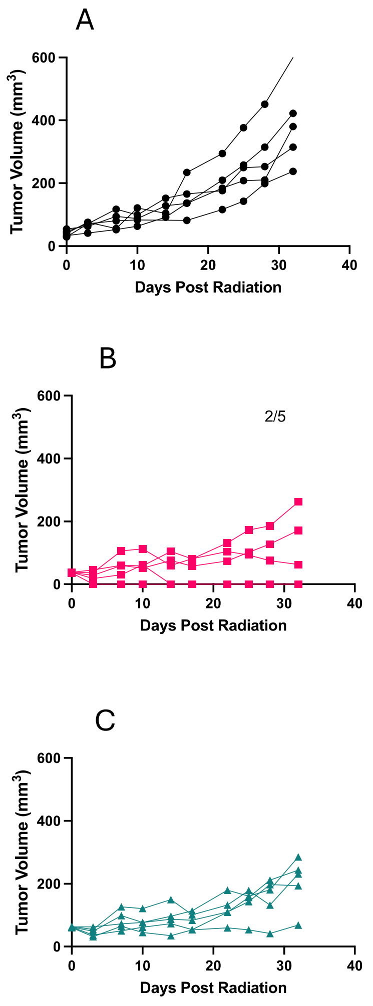

Supplemental Figure 2

Supplemental Figure 2. Systemic effect of CRI in B78 tumor model.

C57BL/6 mice bearing i.d. B78 tumors on the left flank were treated with CRI in which RT and IL-12 injections were applied to the skin in the right flank without the tumor. SrIL-2 and anti-CTLA-4 were given systemically as a part of CRI. (A) no treatment, mean starting tumor volume 40 mm^3; (B) CRI, mean starting tumor volume 37 mm^3; (C) CRI, mean starting tumor volume 62 mm^3.

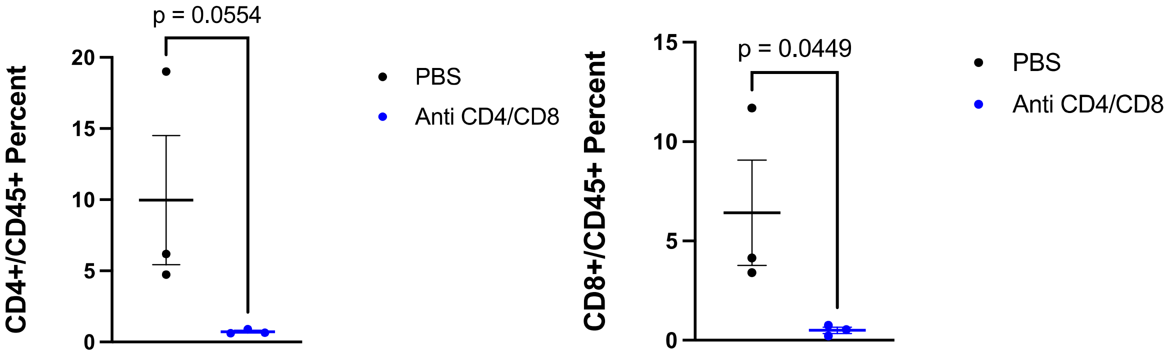

Supplemental Figure 3

Supplemental Figure 3. Depletion of CD4 and CD8 T cells following injection of anti-CD4 and anti-CD8 antibodies.

C57BL/6 mice bearing B78 tumors were injected intraperitoneally with 200mcg of anti-CD4 and 100 mcg of anti-CD8 depleting antibodies in 500 mcl of PBS on Day 0. Tumors were harvested 3 days later and tumor infiltrating lymphocytes were analyzed using flow cytometry. Percentages of $CD4^+$ T cells and $CD8^+$ T cells out of total number of $CD45^+$ cells are shown. Anti-CD4/CD8 treatment induced 92.7% depletion of $CD4^+$ T cells (10% in PBS treated mice vs. 0.73% in anti-CD4/CD8 treated mice) and 92.2% depletion of $CD8^+$ T cells (6.4% in PBS treated mice vs. 0.5% in anti-CD4/CD8 treated mice, mean values of n=3).

A

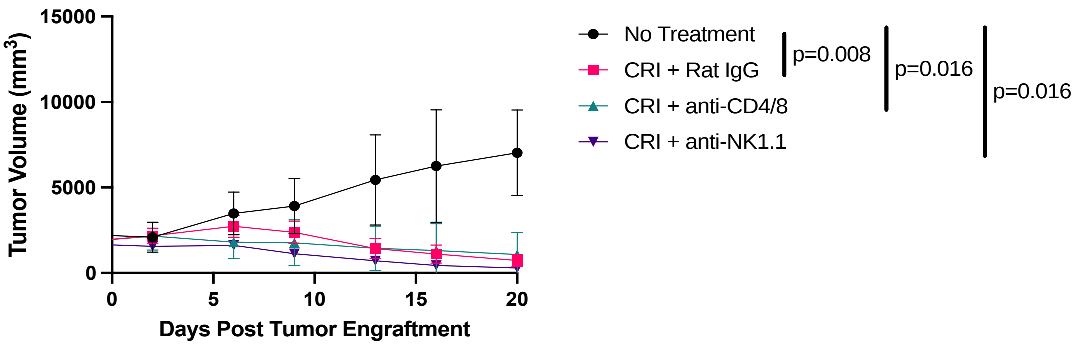

B

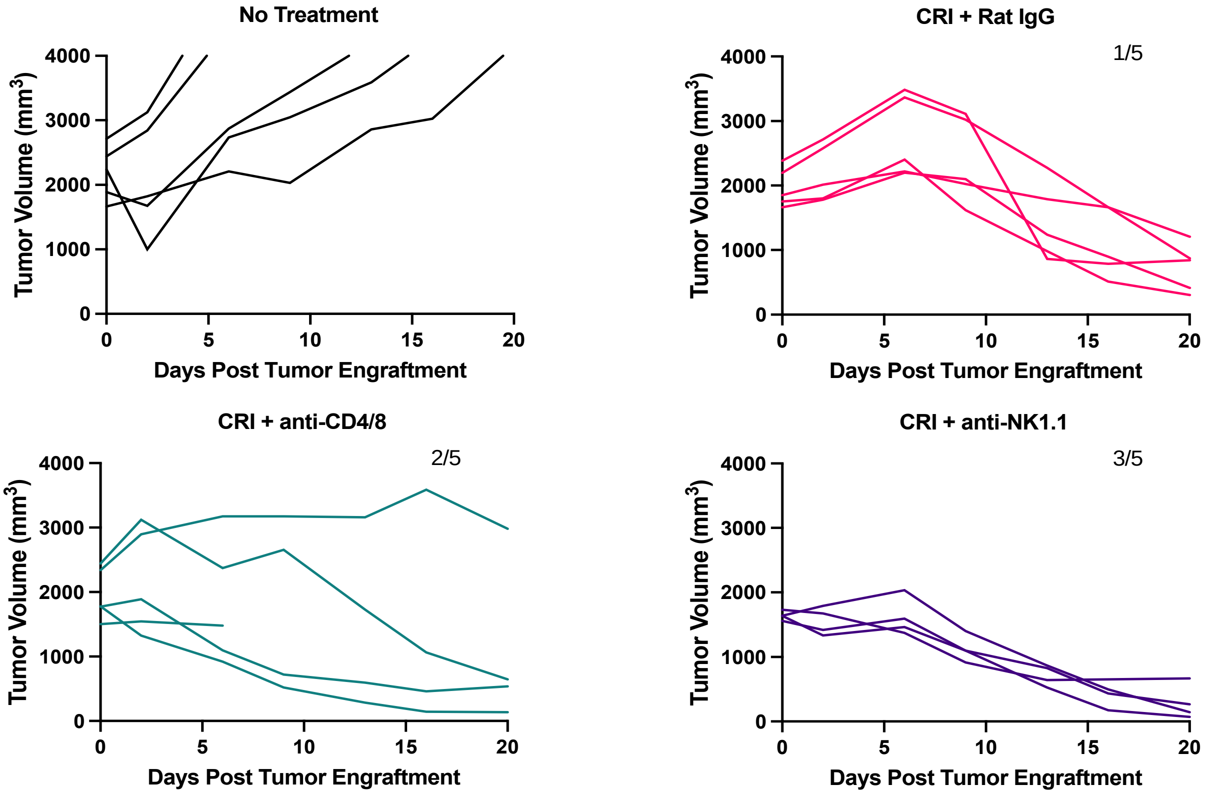

Supplemental Figure 4

Supplemental Figure 4. Role of T cells and NK cells in CRI-induced antitumor effect in B78 melanoma model.

(A) Mean +/- SEM of tumor volumes and (B) individual mouse tumor curves in mice bearing a single B78 tumor are shown following Rat IgG, anti-CD4 + anti-CD8 mAb and anti-NK mAb during CRI treatment, or no treatment.

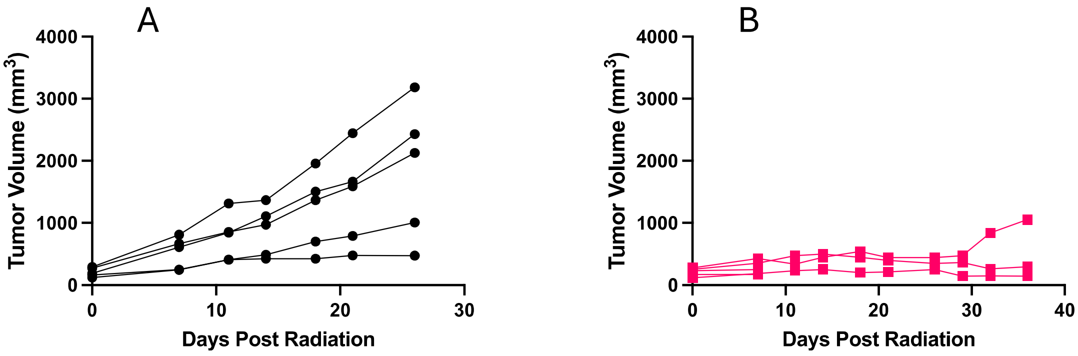

Supplemental Figure 5.

Supplemental Figure 5. Systemic effect of CRI in CT26 tumor model.

Balb/c mice bearing a single i.d. CT26 tumor on the left flank were treated with CRI in which RT and IL-12 injections were applied to the skin in the right flank (normal skin/no tumor implanted). SrIL-2 and anti-CTLA-4 were given systemically as a part of CRI. (A) No treatment, mean starting tumor volume 207 mm^3; (B) CRI, mean starting tumor volume 210 mm^3; none of these mice became tumor-free.

Figure 3A (B78, d8)

Cells	RT (mean +/- SD)	CRI (mean +/- SD)
CD4	13.84 +/- 11.48	5.05 +/- 5.062
CD8	5.863 +/- 6.717	9.713 +/- 8.686
Tregs	28.63 +/- 8.879	19.25 +/- 10.89
CD8:Tregs	4.025 +/- 7.452	30.52 +/- 33.91

Figure 3B (B78, d12)

Cells	RT (mean +/- SD)	CRI (mean +/- SD)
CD4	12.03 +/- 11.26	25.72 +/- 14.7
CD8	6.5 +/- 6.426	23.57 +/- 9.2
Tregs	19.5 +/- 5.928	2.4 +/- 1.667
CD8:Tregs	3.175 +/- 1.442	59.03 +/- 33.09

Figure 5A (MC38, d8)

Cells	RT (mean +/- SD)	CRI (mean +/- SD)
CD4	5.223 +/- 1.946	0.6857+/- 0.4562
CD8	3.483 +/- 2.929	7.943 +/- 10
Tregs	51.83 +/- 9.109	29.57 +/- 11.27
CD8:Tregs	1.183 +/- 0.5269	39.9 +/- 28.48

Figure 5B (MC38, d12)

Cells	RT (mean +/- SD)	CRI (mean +/- SD)
CD4	11.98 +/- 6.309	7.388 +/- 8.35
CD8	12 +/- 8.856	34.38 +/- 19.09
Tregs	29.63 +/- 13.61	13.03 +/- 12.37
CD8:Tregs	4.3 +/- 3.713	304.7 +/- 405.9

Supplemental Table 1. Means +/- SD of Flow experiments in Figures 3A,B and 5A,B.

Percentages are presented for CD4, CD8 and Treg cells. Ratios are presented for CD8:Tregs.
